# Vagus nerve signal has an inhibitory influence on the development of peritoneal metastasis in murine gastric cancer

**DOI:** 10.1038/s41598-024-58440-w

**Published:** 2024-04-03

**Authors:** Yurie Futoh, Hideyo Miyato, Hironori Yamaguchi, Misaki Matsumiya, Rei Takahashi, Yuki Kaneko, Yuki Kimura, Hideyuki Ohzawa, Naohiro Sata, Joji Kitayama, Yoshinori Hosoya

**Affiliations:** 1 Department of Surgery, Division of Gastroenterological, General and Transplant Surgery, Jichi Medical University, Shimotsuke, Japan; 2 Department of Clinical Oncology, Jichi Medical University Hospital, Yakushiji 3311-1, Shimotsuke, Tochigi 329-0498 Japan; 3 Center for Clinical Research, Jichi Medical University Hospital, Shimotsuke, Japan

**Keywords:** Cancer, Cancer microenvironment, Gastrointestinal cancer, Tumour immunology

## Abstract

The vagus nerve is the only pathway for transmitting parasympathetic signals between the brain and thoracoabdominal organs, thereby exhibiting anti-inflammatory functions through the cholinergic anti-inflammatory pathway. Despite often being resected during lymph node dissection in upper gastrointestinal cancer surgery, the impact of vagotomy on postoperative outcomes in gastric cancer patients remains unclear. Sub-diaphragmatic vagotomy was performed on C57BL/6 mice. Three weeks later, syngeneic murine gastric cancer cell line YTN16P was injected into the peritoneal cavity, and the number of peritoneal metastases (PM) on the mesentery and omentum compared with control mice. The phenotypes of immune cells in peritoneal lavage and omental milky spots one day after tumor inoculation were analyzed using flow cytometry and immunohistochemistry. Intraperitoneal transfer of 3 × 10^5^ YTN16P significantly increased the number of metastatic nodules on the mesentery in the vagotomy group compared to the control group. The omental metastasis grade was also significantly higher in the vagotomy group. Phenotypic analysis of immune cells in peritoneal lavage did not reveal significant differences after vagotomy. However, vagotomized mice exhibited a notable increase in milky spot area, with a higher presence of cytokeratin(+) tumor cells, F4/80(+) macrophages, and CD3(+) T cells. Vagus nerve signaling appears to regulate the immune response dynamics within milky spots against disseminated tumor cells and inhibits the development of PM. Preserving the vagus nerve may offer advantages in advanced gastric cancer surgery to reduce peritoneal recurrence.

## Introduction

Gastric cancer ranks as the third leading cause of cancer-related mortality globally despite being the sixth most prevalent malignancy^1,2^. Among the various forms of advanced gastric cancer, peritoneal metastases (PM) represent the most formidable threat to patient survival, with a dismal prognosis^3–5^. Even following curative surgery, nearly half of patients with advanced gastric cancer displaying serosal involvement (T4) experience peritoneal recurrences^6–8^. Thus, the implementation of intensive treatments aimed at mitigating peritoneal recurrence has a pivotal importance for improving the outcome of patients with advanced gastric cancer.

The development of peritoneal metastases is a multifaceted process, encompassing stages such as the detachment of tumor cells from the primary tumor, their adhesion to the peritoneal surface, subsequent invasion into the submesothelial space, and culminating in tumor growth through angiogenesis^9,10^. Notably, immune evasion by disseminated tumor cells within the peritoneal milieu is recognized as a critical event in the progression of PM. The peritoneal cavity harbors a distinctive immune environment that markedly differs from the systemic circulation^11,12^. There are many free-floating immune cells with various phenotypes which sustain peritoneal homeostasis and prevent local inflammation^11,13,14^. Importantly, alterations in the phenotype of these immune cells have been reported in patients with PM^15^. Within this context, "milky spot" cellular aggregates featuring a central core of B cells enveloped by T cells, myeloid cells, and fibroblastic stromal cells embedded in adipocytes beneath the mesothelial cell layer in the omentum^16,17^, play a central role in regulating local immune responses to inflammatory stimuli and cancer ^11,14,18^. Intriguingly, milky spots are known to serve as primary implantation sites for tumor cells in PM, implying that immune cells within milky spots may predominantly exert immunosuppressive functions^19–22^.

The vagus nerve is the tenth cranial nerve and descends from various sublocations within the brain medulla. It innervates multiple visceral organs, including the heart, lung, and abdominal digestive organs. Significantly, the vagus nerve transmits peripheral inflammation to the brain via afferent nerve fibers and subsequently generates signals to suppress the overwhelming inflammation through efferent fibers, a phenomenon known as the cholinergic anti-inflammatory pathway (CAIP) ^23,24^. Although the intricate molecular mechanisms underlying CAIP remain incompletely elucidated, the vagus nerve profoundly influences cardiovascular, neuroendocrine, and immunological systems through multiple neurotransmitters^25–28^. Furthermore, epidemiological investigations have indicated a correlation between heart rate variability which reflects vagus nerve activity, and the prognosis of cancer patients particularly those in advanced stages^29,30^. This observation suggests that CAIP may also exert a significant impact on tumor progression. Indeed, animal studies in the last century showed that vagotomy promoted the carcinogenesis of gastrointestinal cancers^31–33^. These findings strongly implicate a causal relationship between impaired vagus nerve activity and metastasis formation of abdominal cancer, raising the possibility of a significant association between vagotomy and peritoneal recurrence.

In the context of operative procedures for upper gastrointestinal tract cancer, the partial or complete transection of the vagus nerve is frequently undertaken to achieve comprehensive lymph node dissection. However, the effects of vagotomy on the postoperative outcomes of patients with gastric cancer have not been thoroughly explored. Therefore, the present study was designed to investigate the impact of vagotomy on the development of PM, with a particular focus on peritoneal immunity utilizing a syngeneic murine gastric cancer model.

## Material and method

### Cell line and cell culture

The study utilized the mouse gastric cancer cell line YTN16 which was induced through oral administration of N-Methyl-N-nitrosourea (MNU) in p53 heterozygous knockout C57BL/6 mice. This particular cell line was generously provided by Dr. S. Nomura from Tokyo University, Japan. A highly metastatic subline YTN16P was established through an in vivo selection method involving repeated intraperitoneal administration of the metastatic subline of YTN16^34^. Culturing of these cells was conducted in Dulbecco’s Modified Eagle Medium (DMEM) supplemented with 10% fetal bovine serum (FBS; Sigma, St. Louis, MO, USA), 50U/mL penicillin, 50 mg/mL streptomycin (Life Technologies, Grand Island, NY, USA), 2.5 µg/mL Plasmocin prophylactic (InvivoGen, San Diego, USA), and an additional 2 µM/mL L-Glutamine (G7513 200 mM; Sigma). The cell culture was maintained on Collagen-I coated dishes within a 5% CO2 incubator set at 37 °C.

### Isolation of cells from peritoneal cavity, blood or spleen of mice

Cold PBS (10 ml) was injected into the peritoneal cavity of euthanized mice. Peritoneal lavage was collected following abdominal massage and filtered through a 40 µm nylon mesh and dissociated into single-cell suspension. Spleens were fragmented into pieces using the top rubber of an inner cylinder of a 5 ml syringe on cell strainers (FALCON, Aichi, Japan). The fragments were filtered to obtain a single-cell suspension. Blood cells were collected from inferior vena cava and mixed with citrate buffer for prevention of coagulation. Red blood cells were lysed with RBC Lysis Buffer and removed from suspension of splenocytes or blood cells by washing. These single cell suspensions were used for flowcytometric analysis.

### Flow cytometry

Cells were obtained from the single-cell suspension of mouse tissues and stained with FVS reagents in PBS to label deceased cells. Following a PBS wash, the cells underwent a 10-min incubation with FcR Blocking Reagent (Miltenyi Biotec) to prevent nonspecific antibody binding. Subsequently, the cells were exposed to fluorescently labeled antibodies, as specified in Supplementary Table 1, for 30 min. For intracellular antigen detection, the cells were permeabilized using the Cytofix/Cytoperm™ Fixation/Permeabilization Kit (BD Biosciences, USA) in accordance with the manufacturer's guidelines, and intracellular antigens were probed with the antibodies listed in Supplementary Table 1, also for 30–60 min. Following the staining process, the cells were washed, and data acquisition was performed using an LSR Fortessa™ X-20 instrument (BD, San-Jose, CA USA), with subsequent analysis using Flow Jo™ software (BD, San-Jose, CA USA). CD45 expression was utilized to identify blood cells, with CD11b(+) cells classified into the myeloid cell lineage, CD3(+) cells into the T lymphocyte lineage, and CD19(+) cells into the B lymphocyte lineage. Within the myeloid cell lineage, CD11b^high^F4/80^high^MHCII^low^ was categorized as large peritoneal macrophage (LPM), CD11b^mid^F4/80^low^MHCII^high^ as small peritoneal macrophage (SPM), CD11b^mid^Ly6G/Ly6C^low^CCR2^high^ as classical monocyte (inflammatory monocytes), CD11b^mid^Ly6G/Ly6C^low^CX3CR1^high^ as non-classical monocyte (patrolling monocytes), and CD11b^mid^Ly6G/Ly6C^high^ as granulocytes. In the T lymphocyte lineage, cells were classified as CD4(+) T cells, CD8(+) T cells, and regulatory T cells (Treg) with the phenotype of CD4(+) CD25(+) Foxp3(+).

### Animal experiment

Female C57BL/6N mice were obtained from CLEA Japan Inc. in Tokyo, Japan, and were housed in controlled environments with constant temperature and humidity, following a twelve-hour day-night cycle. The experiments commenced when the mice had adapted to their new surroundings, typically at 6–9 weeks of age. Isoflurane anesthesia was administered (induction at 3%, maintenance at 1.5–2% of minimum alveolar concentration) prior to laparotomy, which involved an upper midline incision.

**Figure 1:**
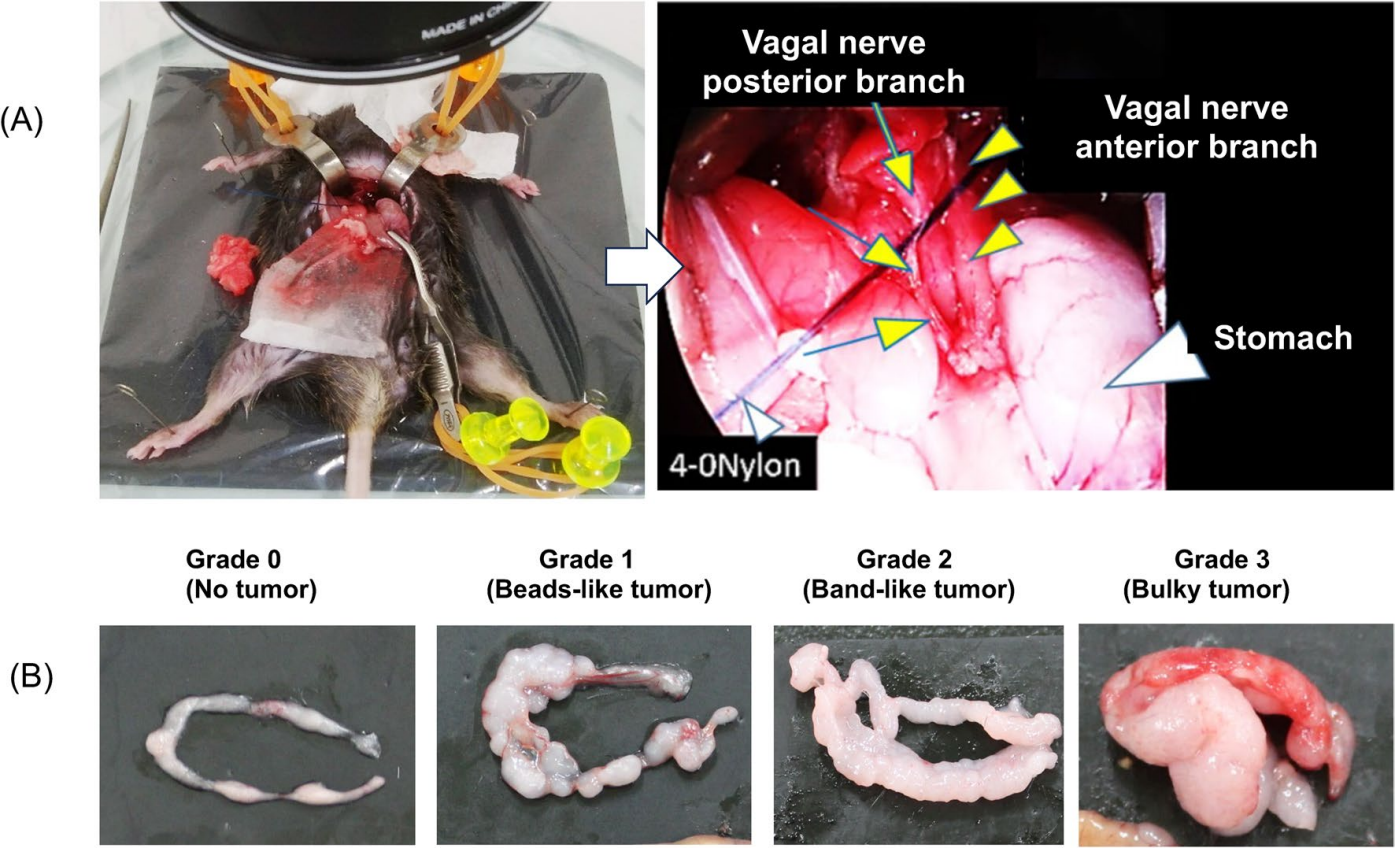
(**A**) Subdiaphragmatic Vagotomy in Mice under a Stereomicroscope. The arrows and arrowheads represent the anterior and posterior branches of the vagus nerve, respectively. (**B**) Macroscopic Assessment of Omental Metastasis. Grade 1 was defined when the omentum exhibited a bead-shaped appearance. Omental metastasis was categorized as grade 2 when the omentum was completely replaced by metastatic lesions, resulting in a band-like shape, and as grade 3 when it exhibited a mass-like shape.

Under a stereomicroscope (10 × magnification, Nikon SMZ800N, Tokyo, Japan), the bilateral trunks of the vagus nerve along with the liver branch were identified running along the abdominal esophagus (Fig. 1A). In the vagotomy groups (VxPP), approximately 3–5 mm of vagus nerves were resected (sub-diaphragmatic vagotomy), followed by pyloroplasty. Pyloroplasty involved cutting the serosa and muscle of the pylorus with a scalpel and suturing the serosal layer with 6–0 nylon (AR13 6-0N; Natsume, Japan). Subsequently, the abdominal wall was closed using 4–0 nylon (A10 4-0N; Natsume) in a layered fashion. In the sham operation groups (PP), the vagus nerves were exposed (vagus-nerve preservation) and pyloroplasty was carried out.

Three weeks following surgery, YTN16P cells (1 × 10^6^ or 3 × 10^5^ cells, 500 µL) were intraperitoneally (IP) injected into the C57BL/6N mice, and PM was evaluated based on macroscopic nodules on the mesentery and omentum. Omental metastases were categorized into four grades according to macroscopic morphology (Grade 0: No tumor, Grade 1: Bead-like tumors, Grade 2: Band-like tumors, and Grade 3: Bulky tumors) as illustrated in Fig. 1B. Two investigators blinded to the treatment groups independently assessed the metastases, and the average values of their evaluations were recorded. All procedures were conducted in compliance with the guidelines approved by the Animal Care Committee of Jichi Medical University (Approval Numbers: 20013-02 and 23022-01) and were performed in accordance with ARRIVE guidelines.

### Immunohistochemistry of mouse omentum

The excised mouse omentum was fixed in formalin and subsequently embedded in paraffin. The resulting tissue blocks were then sliced into 4-μm thick sections for immunohistochemistry. These tissue sections placed on glass slides were subjected to baking at 60 °C and deparaffinization through sequential immersion in xylene and ethanol. To block endogenous peroxidase activity, treatment with hydrogen peroxide in methanol was carried out. Antigen retrieval was achieved by microwave heating in citrate buffer. Following antigen retrieval, the tissue sections underwent a 30-min incubation with Blocking One Histo to prevent nonspecific antibody binding. Subsequently, the sections were incubated with antibodies against CD3 (1:150), CD19 (1:1000), Ly-6G/Ly-6C (1:50), F4/80 (1:100), or pan Cytokeratin (1:200) in humid chambers at room temperature for 60 min. After thorough washing with PBS-T (PBS containing 500 µl of TWEEN20 in 1L), the sections were incubated with a secondary antibody conjugated with peroxidase at room temperature for 30 min. Following another round of washing, the presence of antigens was visualized by the addition of the enzyme substrates 3,3'-Diaminobenzidine (DAB) or 3-Amino-9-ethylcarbazole (AEC). Finally, the sections were counterstained with Meyer’s hematoxylin. The specimens were examined under microscope and number and size of milky spots were assessed using ImageJ Fiji (National Institutes of Health, Bethesda, MD). To assess cell density, positively stained cells in five randomly chosen milky spots were independently counted by two investigators, and the average cell counts within 1 mm^2^ area were calculated.

### Statistical analysis

Details regarding the specific statistical analyses conducted for each experiment were provided within the respective Figure legends. The data were presented as means ± standard deviation or median (range), and statistical analyses were performed using Mann–Whitney U tests in Graph Pad Prism 8 Software (SanDiego, CA, USA) with a significance threshold set at P < 0.050.

### Human and animal rights

The protocol of animal study was approved by Institution Review Board of the Animal Care Committee of Jichi Medical University (approval no. 20013).

## Results

### Change in body weight and systemic immunity after vagotomy

**Figure 2:**
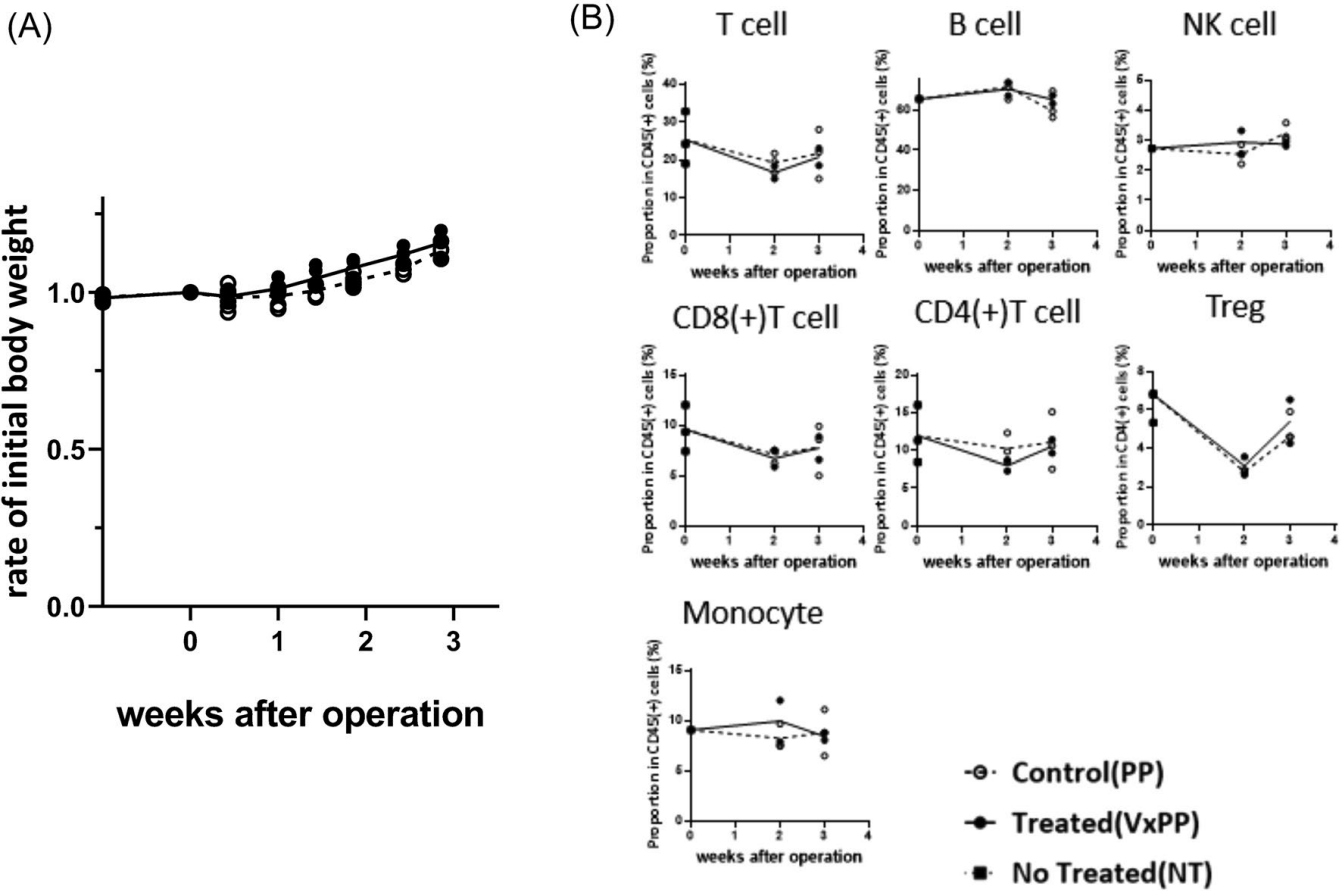
Change in body weight (**A**) and phenotype of splenocytes (**B**) after vagotomy. Splenocytes were isolated from mice 2 and 3 weeks after surgery, and their phenotypes were assessed using flow cytometry.

We initially assessed postoperative recovery in mice undergoing surgery. Body weight began to increase after 2 weeks, with no significant differences between vagotomy and pyloroplasty (VxPP) and control (PP) groups (Fig. 2A). To evaluate the immune system, we examined cellular composition in the spleen at 2- and 3-weeks post-surgery. At 2 weeks, proportions of CD4(+) and CD8(+) T cells in CD45(+) splenocytes, particularly CD4(+) CD25(+) Foxp3(+) T regs, tended to decrease but returned to pre-surgery levels by 3 weeks. B cells, NK cells, and Ly6C(+) monocytes/macrophages showed no significant changes from preoperative values. No significant differences were observed between the two groups during these periods (Fig. 2B).

### Enhanced peritoneal metastasis after vagotomy

**Figure 3:**
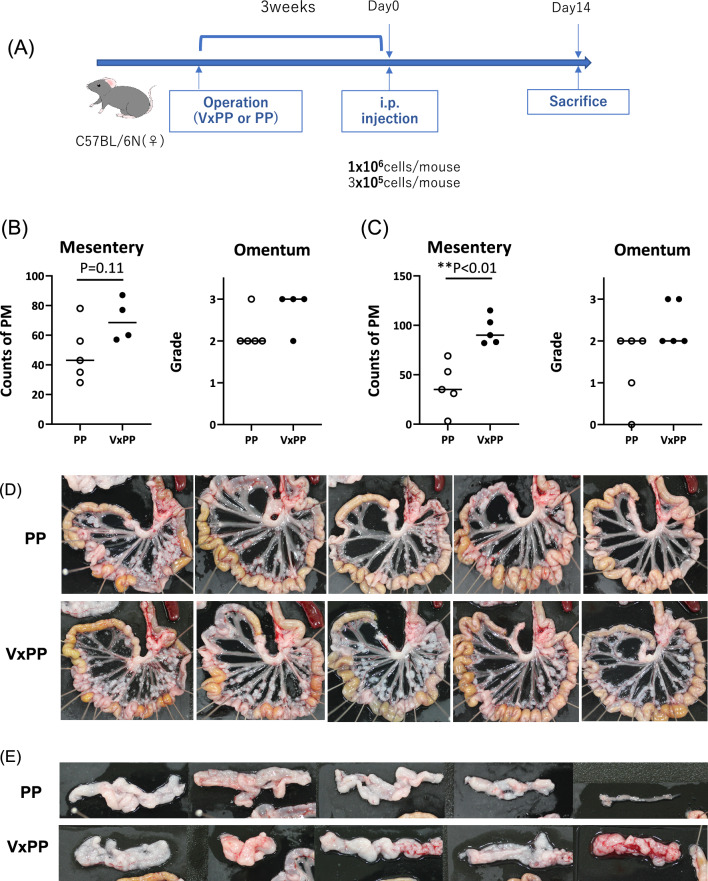
(**A**) Experimental design for the evaluation of peritoneal metastasis (PM). Three weeks following surgery, C57BL/6N mice were intraperitoneally injected with 1 × 10^6^ (**B**) or 3 × 10^5^ (**C**–**E**). YTN16P cells in 500 µL of HBSS. PM was assessed based on macroscopic nodules on the mesentery and omentum. Omental metastases were categorized into four grades according to macroscopic morphology, as illustrated in Fig. 1B. P-values were calculated using the Mann–Whitney U test.

At 3 weeks post-surgery when systemic immunity was presumed to have mostly returned to preoperative conditions, we injected YTN16P tumor cells into the peritoneum and examined PM formation in the omentum and mesenteries after 2 weeks (Fig. 3A). When transferring 1 × 10^6^ tumor cells, macroscopic nodules on the mesentery were slightly more numerous in the VxPP group compared to the PP group, although the difference was not significant (PP: M = 43(28–78) vs VxPP: M = 68.5(57–87) p = 0.11, n = 5, 4). In all mice, the omentum was mostly replaced by tumors with band-like or bulky metastases (Fig. 3B, Supplementary Fig. 2). However, when transferring 3 × 10^5^ tumor cells, the number of metastatic nodules on the mesentery significantly increased in the vagotomy group (PP: M = 35(3–69) vs VxPP: M = 90(82–115), p = 0.008, n = 5) (Fig. 3C,D). The grade of macroscopic appearance of omental metastasis was significantly higher in the VxPP group (Fig. 3C,E).

### Changes in immune cells in blood and abdominal cavity after tumor inoculation

**Figure 4:**
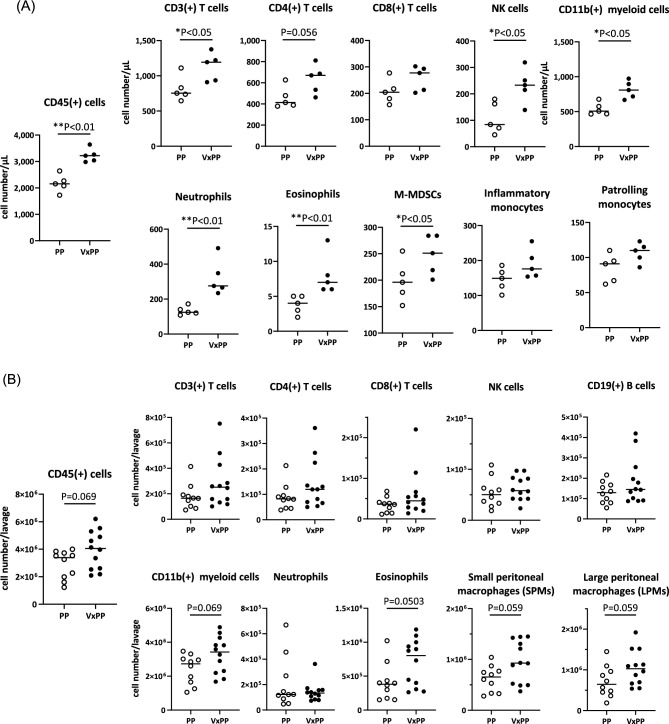
Effects of vagotomy on systemic and local immune cell composition against intraperitoneal tumor challenge. Three weeks following surgery, YTN16P (3 × 10^5^) were intraperitoneally transferred and immune cells were collected from circulating blood and the peritoneal cavity on the following day as described in the Materials and Methods. Their phenotypes were characterized using flow cytometry, and the numbers of each cell type in 1 µl of blood (**A**) or in the entire abdomen (**B**) were calculated. P-values were calculated using the Mann–Whitney U test.

To assess the influence of vagotomy on early immune responses following intraperitoneal (IP) injection of YTN16P, we collected immune cells from both the circulating blood and peritoneal cavity on the day following tumor inoculation, and subsequently characterized these immune cells using flow cytometry with the gating strategy depicted in Supplementary Fig. 1. The mice subjected to vagotomy exhibited a significantly higher number of all cell types compared to the control group. Notably, T cells, NK cells, and myeloid cells especially neutrophils and eosinophils, demonstrated a marked elevation in VxPP group (Fig. 4A). In contrast, no significant differences in the subsets of lymphocytes and myeloid cells were observed between the two groups, although there was a trend towards higher eosinophils and macrophages in the vagotomy group, with marginal differences (Fig. 4B). Among macrophages, the proportions of large peritoneal macrophages (LPM) and small peritoneal macrophages (SPM) did not show the difference between the two groups.

### Changes in milky spots in omental tissues

**Figure 5:**
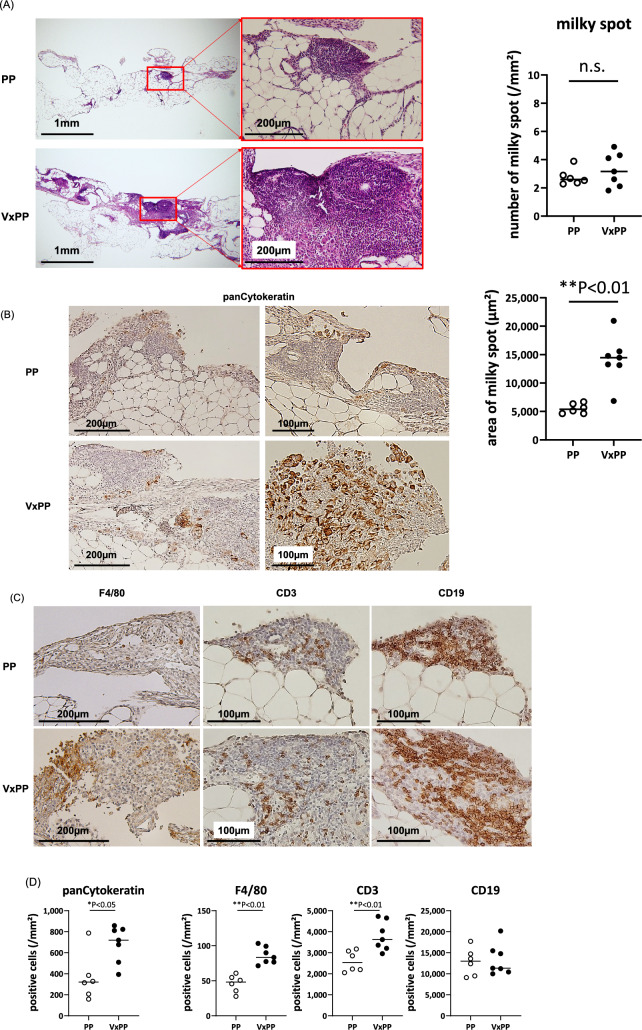
Effects of vagotomy on omental milky spots after intraperitoneal tumor challenge. Three weeks following surgery, YTN16P (3 × 10^5^) were intraperitoneally transferred. Omenta were harvested on the following day and specimens were stained with Hematoxylin–Eosin (**A**) and subjected to immunohistochemistry using specific monoclonal antibodies (**B**,**C**). (**A**) The number and size of milky spots were measured using ImageJ Fiji. (**D**) The density of cytokeratin (+) tumor cells (**B**), as well as F4/80(+) macrophages, CD3(+) T cells, and CD19(+) B cells (**C**), were calculated as described in the Materials and Methods section. P-values were calculated using the Mann–Whitney U test.

We examined the histological changes in omental tissues on the following day after tumor inoculation. Although the number of milky spots did not significantly change in the VxPP group, many milky spots exhibited remarkable enlargement with high cell density while most remained intact in the PP group (Fig. 5A). Specifically, the milky spot area in the VxPP group was approximately five times larger than that in the PP group (PP: M = 5380(4640–6692) µm^2^ vs VxPP: M = 14,460(6844–20,944) µm^2^, p = 0.0012, n = 6,7).

As shown in Fig. 5B,D, immunostaining revealed a significant increase in the density of cytokeratin (+) tumor cells within milky spots of vagotomized mice compared to the PP group (PP: M = 321 (159–787)/mm^2^ vs VxPP: M = 719(394–858)/mm^2^, p = 0.014, n = 6,7). The number of CD3(+) T cells, but not CD19(+) B cells, increased in milky spots of vagotomized mice (CD3(+); PP: M = 2541(2052–3177)/mm^2^ vs VxPP: M = 3627(2957–4731)/mm^2^, p = 0.0047) (Fig. 5C,D). A relatively small number of F4/80(+) macrophages were observed at the periphery of milky spots, and their density also significantly increased in the VxPP group (PP: M = 48.2(27.8–60.8)/mm^2^ vs VxPP: M = 83.4(71.4–103)/mm^2^, p = 0.0012) (Fig. 5C,D).

## Discussion

The vagus nerve represents the exclusive conduit for transmitting parasympathetic signals between the brain and thoracoabdominal organs, thereby exhibiting anti-inflammatory functions that safeguard against the progression of numerous diseases^23–27^. In our study, prior vagotomy significantly increased PM upon intraperitoneal inoculation of gastric cancer cells (YTN16P) in syngeneic mice. Earlier animal studies in the last century demonstrated that vagotomy promotes the development of gastrointestinal cancers such as gastric^31^, colon^32^ and pancreatic^33^ cancers. Recent research also indicates that partial vagotomy increases metastasis in breast and colon cancers^35,36^. Partecke et al. reported that subdiaphragmatic vagotomy exacerbates pancreatic cancer progression involving tumor-associated macrophages (TAM) producing tumor necrosis factor-alpha (TNF-α) in a murine model^37^. Our findings align with their results and propose that vagal activity may play a protective role in inhibiting the formation of PM.

Given the significant impact of vagus nerve signals on abdominal immune responses, we examined immune cell phenotypes in circulating blood and peritoneal washings one day after tumor inoculation. Notably, the enumeration of granulocytes, T cells, and NK cells in the circulating blood demonstrates a pronounced elevation in vagotomized mice. This implies that the absence of afferent vagal signals may trigger an exaggerated systemic immune response against peritoneal tumor cell challenge. However, no significant differences were observed in peritoneal immune cells except for peritoneal macrophage and eosinophil between vagotomized and control mice. In the rat model, it was documented that vagotomy leads to a reduction in the peritoneal resident cell population, fever, and neutrophil migration to the peritoneal cavity following IP injection of LPS. Furthermore, studies have reported that vagal signaling effectively suppresses chemokine release and the influx of neutrophils into the peritoneal cavity in mouse peritonitis models induced by IP injection of Escherichia coli (E. coli)^38^ or zymosan ^39^. These findings collectively suggest that vagal signaling plays a suppressive role in neutrophil-mediated inflammation within the peritoneal cavity. In this study, no substantial increase in neutrophil counts was observed within the peritoneal cavity at early time point. This observation implies the presence of distinct stimuli or mechanisms associated with tumor-related responses as opposed to those elicited by conventional inflammatory stimulants.

On the contrary, prior investigations have established the pivotal role of the vagus nerve in regulating the release of TNFα from peritoneal macrophages, leading to a potential reduction in systemic inflammatory response syndrome^40,41^. Moreover, a previous study demonstrated that intravenous adoptive transfer of peritoneal macrophages obtained from peritoneal lavage following vagus nerve stimulation conferred protection to the kidneys against ischemia–reperfusion injury.^42^ These findings suggest that vagal signaling may exert a significant influence on peritoneal immunity and endow peritoneal macrophages with the capability to attenuate inflammation. Therefore, further functional analysis of how intraperitoneal cells behave might reveal noticeable differences in vagotomized mice.

Conversely, our examination of omental milky spots revealed notable changes resulting from prior vagotomy. The milky spot area exhibited a marked increase at this time point, along with a higher presence of cytokeratin (+) tumor cells. Milky spots are known as primary implantation sites for malignant cells in PM, as documented in previous studies^19–22^. Clark et al. demonstrated that tumor cells within milky spots gain the potential for progressive growth and dissemination within the peritoneal cavity, primarily facilitated by the energy supply from surrounding adipocytes^43^. Collectively, these findings suggest that the observed increase in PM following vagotomy may be attributed to an elevated number of tumor cells colonizing and surviving within milky spots at an earlier time point, underscoring their pivotal role in this phenomenon.

Milky spots are considered conduits for the recruitment of peritoneal fluids into the peritoneal cavity^44,45^, and disseminated tumor cells may be passively transported via the flow of peritoneal fluids^19,46^. However, previous research suggests that the preferential implantation of tumor cells in milky spots may be attributed to chemokines like IL-8 and CXCL12 ^47,48^ or release of neutrophil extracellular traps (NETs) ^49^. Additionally, Etzerodt et al. demonstrated that tissue-resident macrophages in milky spots enhance the malignant potential of disseminated tumor cells, playing a crucial role in the progression of PM^50^. In our study, we observed increased densities of F4/80(+) macrophages and CD3(+) T cells in the milky spots of vagotomized mice, suggesting the possibility that these immune cells with tumor-promoting capacity are elevated in omental milky spots after vagus nerve transection.

Omental milky spots akin to mesenteric lymph nodes constitute a secondary lymphoid tissue that plays a pivotal role in the regulation of peritoneal immunity. The precise mechanism by which vagus nerve signaling modulates the immune response in milky spots against tumors remains unclear. Current evidence points to the synaptic connection of vagus nerves with sympathetic nerves located within the celiac ganglia. Subsequently, these postganglionic nerves establish connectivity with the spleen. In the splenic environment, noradrenaline released from sympathetic nerves exerts its influence on β2 adrenaline receptors present on T cells expressing choline acetyltransferase (ChAT). This interaction ultimately leads to the release of acetylcholine (ACh). Then, ACh in turn functions to suppress the production of TNF-α through activation of the alpha7 nicotinic acetylcholine receptor (a7nAChR) expressed on monocytes/macrophages, lymphocytes, and other cytokine-producing cells^40,51,52^. A parallel mechanism known for its role in suppressing systemic inflammation has been reported to operate within the mesenteric lymph nodes^53^. Moreover, vagotomy has been reported to abolish the anti-inflammatory impact on tumor-infiltrating macrophages (TAM) and promote the production of proinflammatory cytokines, thereby altering the microenvironment of lung and pancreas to facilitate tumor progression^35,37^. A recent immunohistochemical study has also revealed a substantial number of sympathetic nerve fibers in omental milky spots in adult humans^54^. Since the neuro-immune response in spleen and mesenteric lymph nodes is dependent on sympathetic nerve stimulation^40,51–53^, it is possible that vagotomy could change omental milky spots in a same mechanism.

The a7nAChR subunits are also expressed in many tumor cells allowing them to be directly modulated in various aspects of their properties, including proliferation, apoptosis, invasion, and angiogenesis ^55,56^. Therefore, the attenuation of the CAIP signal following vagotomy could potentially exert a direct stimulatory effect on the survival and proliferation of tumor cells sequestered within milky spots.

In summary, vagotomy resulted in an increased deposition of tumors in the milky spots shortly after intraperitoneal inoculation of gastric cancer cells, ultimately leading to the augmentation of PM formation. Prior research has established that the preservation of the vagus nerve during surgery for early gastric cancer yields benefits such as a reduction in the incidence of gallstones, postoperative diarrhea, and bile reflux^57–59^. Conversely, for surgery targeting advanced gastric cancer, preserving the vagus nerve is considered impractical due to oncological safety, namely to prevent the risk of insufficient lymph node dissection. Nevertheless, our findings suggest the potential merits of vagus nerve-preserving gastrectomy even in cases of advanced gastric cancer because the absence of postoperative vagal immunomodulation could contribute to heightened aggressiveness in peritoneal recurrences. In this investigation, however, we conducted analysis to assess the impact of subdiaphragmatic vagotomy in the absence of gastrectomy. The inability to perform gastrectomy in mouse model constitutes a limitation in drawing definitive conclusions on this matter. Further research using larger animal models with vagus nerve preservation during gastrectomy is essential to replicate clinical scenarios accurately.

### Supplementary Information

Supplementary Information 1.

Supplementary Information 2.

## Data Availability

The datasets used and/or analyzed during the current study available from the corresponding author on reasonable request.
