# Supplementary material for: Vagus nerve signal has an inhibitory influence on the development of peritoneal metastasis in murine gastric cancer
Source: Sci Rep. 2024 Apr 3;14:7832. doi: 10.1038/s41598-024-58440-w (PMC10991300; doi:10.1038/s41598-024-58440-w)

Supplementary Figure 1 (A)

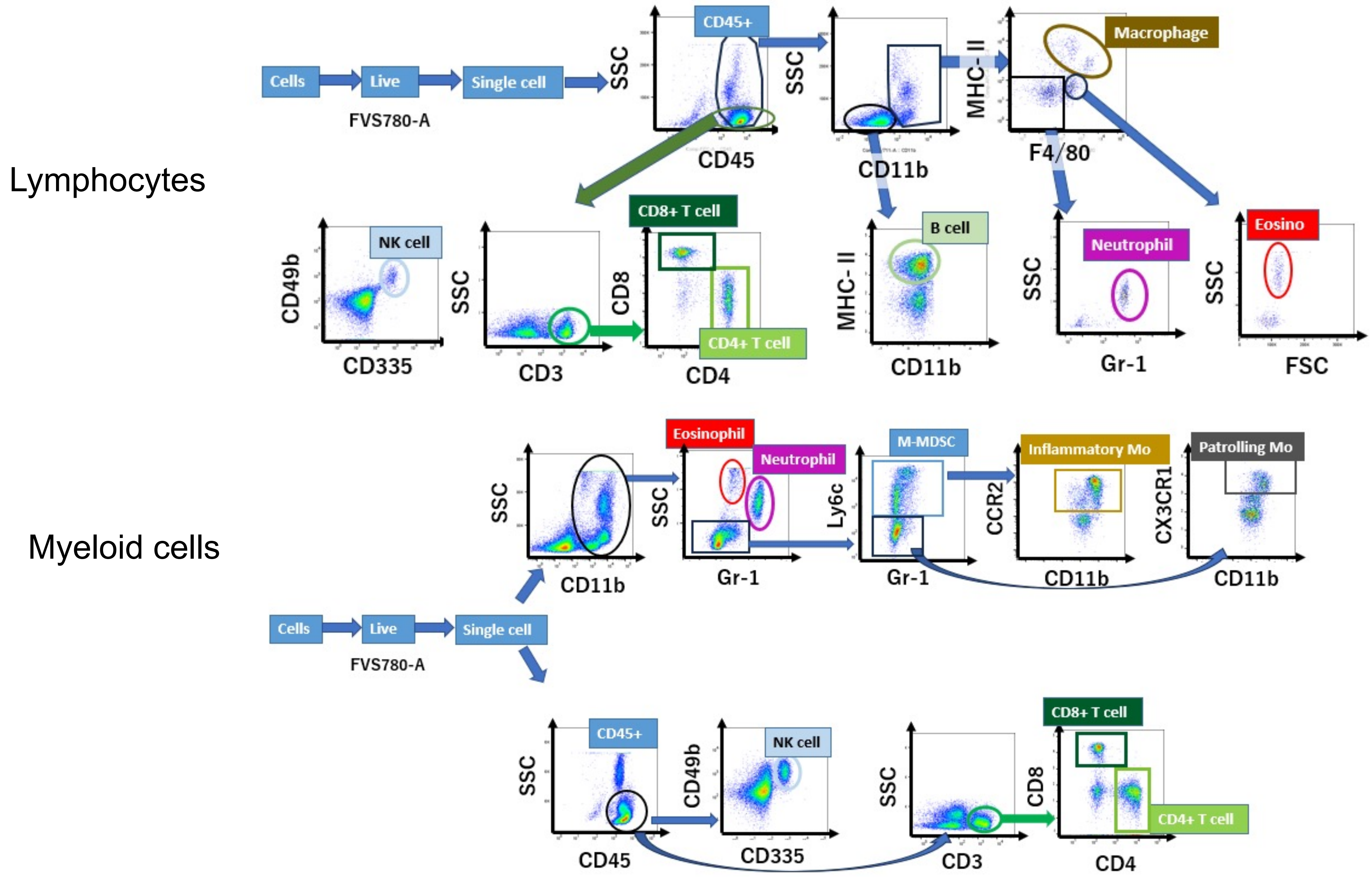

Supplementary Figure 1 (B)

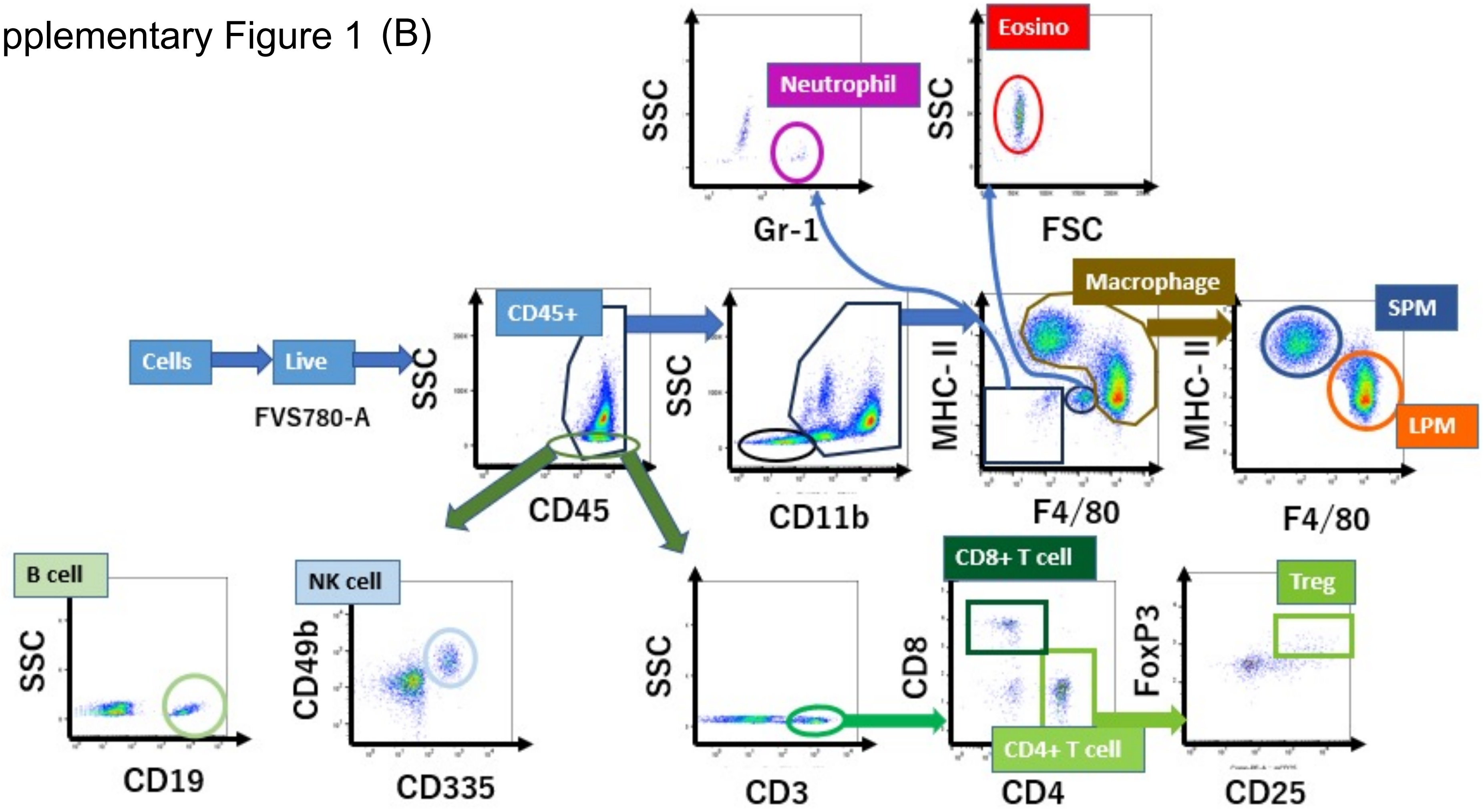

Supplementary Figure1. Gating strategy of flowcytometric analysis of cells derived from circulating blood (A) and abdominal cavity (B)

Supplementary Figure 2

C57BL/6N mice were intraperitoneally injected with 1×10^6 YTN16P cells in 500 μL of HBSS. PM was assessed based on macroscopic nodules on the mesentery and omentum.

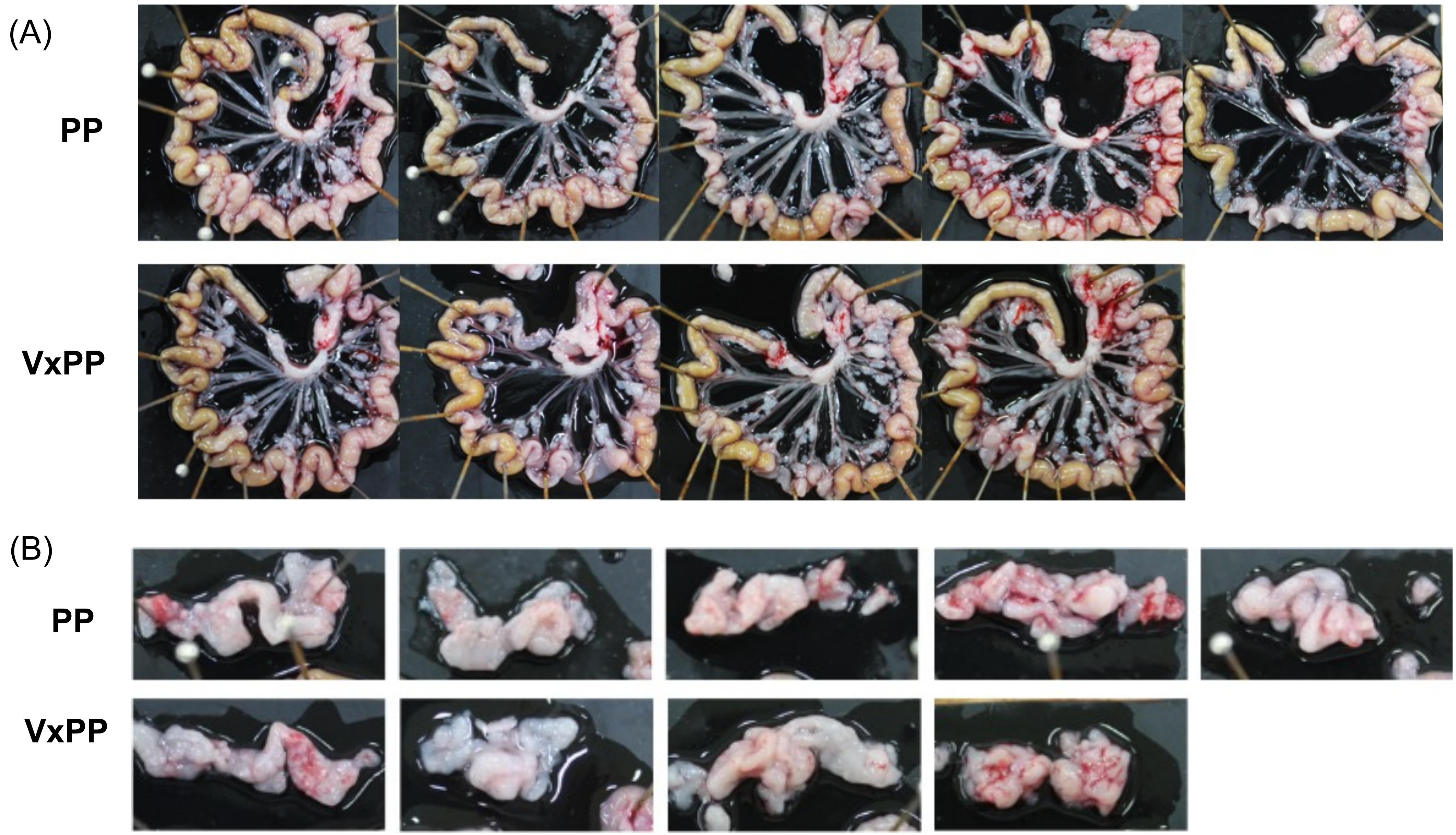

Supplement: Supplementary file 1 — Supplementary Information 1. [file 41598_2024_58440_MOESM1_ESM.pdf]
