# Supplementary material for: Vagus nerve signal has an inhibitory influence on the development of peritoneal metastasis in murine gastric cancer
Source: Sci Rep. 2024 Apr 3;14:7832. doi: 10.1038/s41598-024-58440-w (PMC10991300; doi:10.1038/s41598-024-58440-w)
Supplement: Supplementary file 2 — Supplementary Information 2. [file 41598_2024_58440_MOESM2_ESM.docx]

**Supplementary Table 1. Antibodies and reagents**

| REAGENT | SOURCE | IDENTIFIER |
| --- | --- | --- |
| **Antibody (Flow cytometry)** |  |  |
| CD3 Alexa Fluor® 700, 17A2 | Bio Legend | Cat# 100216 |
| CD4 FITC, GK1.5 | Bio Legend | Cat# 100406 |
| CD4 BV421, GK1.5 | Bio Legend | Cat# 100443 |
| CD8a APC, 53-6.7 | Bio Legend | Cat# 100711 |
| CD8a PE, 53-6.7 | Bio Legend | Cat# 100708 |
| CD11b APC, M1/70 | Bio Legend | Cat# 101212 |
| CD11b BV480, M1/70 | BD | Cat# 566117 |
| CD11b BV711, M1/70 | BD | Cat# 563168 |
| CD19 PE, 6D5 | Bio Legend | Cat# 115507 |
| CD25 PE, PC61 | Bio Legend | Cat# 102008 |
| CD45 FITC, 30-F11 | Bio Legend | Cat# 103108 |
| CD45 BUV395, 30-F11 | BD | Cat# 565967 |
| CD49b FITC, DX5 | Bio Legend | Cat# 108906 |
| CD86 PE, GL-1 | Bio Legend | Cat# 105008 |
| CD192(CCR2) BUV395, 475301 | BD | Cat# 747972 |
| CD206 BV421, C068C2 | Bio Legend | Cat# 141717 |
| CD274 (B7-H1, PD-L1) APC, 10F.9G2 | Bio Legend | Cat# 124312 |
| CD279 (PD-1) BV421, 29F.1A12 | Bio Legend | Cat# 135221 |
| CD335 APC, 29A1.4 | Bio Legend | Cat# 137608 |
| CX3CR1 PE, SA011F11 | Bio Legend | Cat# 149006 |
| F4/80 BUV395, T45-2342 | BD | Cat# 565614 |
| FOXP3 BV421, MF-14 | Bio Legend | Cat# 126419 |
| GATA6 PE, D61E4 | Cell Signaling | Cat# 26452S |
| I-A/I-E (MHC class II) APC, M5/114.15.2 | Bio Legend | Cat# 107614 |
| I-A/I-E (MHC class II) PE, M5/114.15.2 | Bio Legend | Cat# 107607 |
| Ly-6C FITC, HK1.4 | Bio Legend | Cat# 128006 |
| Ly-6C PE, HK1.4 | Bio Legend | Cat# 128008 |
| Ly-6G/Ly-6C (Gr-1) BV421, RB6-8C5 | Bio Legend | Cat# 108445 |
| Tim4 PE, RMT4-54 | Bio Legend | Cat# 130006 |
| Rabbit IgG PE, DA1E | Cell Signaling | Cat# 5742S |
| Rat IgG2aκ BV421, RTK2758 | Bio Legend | Cat# 400549 |
| Rat IgG2aκ PE, RTK2758 | Bio Legend | Cat# 400508 |
| Rat IgG2bκ APC, RTK4530 | Bio Legend | Cat# 400612 |
| Rat IgG2bκ BV421, RTK4530 | Bio Legend | Cat# 400655 |
| Rat IgG2bκ BUV395, R35-38 | BD | Cat# 563560 |
| Rat IgG1λ PE, G0114F7 | Bio Legend | Cat# 401906 |
| Mouse IgG2aκ PE, MOPC-173 | Bio Legend | Cat# 400212 |
| BD Horizon™ Fixable Viability Stain 780 | BD | Cat# 565388 |
| PKH26 Red Fluorescent Cell Linker Kit for Phagocytic Cell Labeling | Sigma Aldrich | Cat#PKH26PCL |
| **Antibody (Immunohistochemistry, IHC)** |  |  |
| Anti CD3e, SP7, rabbit IgG | Thermo Fisher | Cat# MA5-14524 |
| AntiMouseCD8a, 4SM15, rat IgG2aλ | Invitrogen | Cat# 14-0808-82 |
| AntiMouseCD19, EPR23174-145, rabbit IgG | Abcam | Cat# ab245235 |
| AntiMouseF4/80, BM8, rat IgG2aκ | Bio Legend | Cat# 123102 |
| AntiMouseLy6G/Ly6C, RB6-8C5, rat IgG2bκ | Invitrogen | Cat# 14-5931-85 |
| Anti pan-Cytokeratin, KRT1877R, rabbit IgG | Abcam | Cat# ab234297 |
|  |  |  |
| **Reagent** |  |  |
| FcR Blocking Reagent, mouse | Miltenyi Biotec | Cat#130-092-575 |
| BD Horizon™ Brilliant Stain Buffer Plus | BD | Cat# 566385 |
| BD Cytofix™ Fixation Buffer | BD | Cat# 554655 |
| BD Cytofix/Cytoperm™ Fixation/Permeabilization Kit | BD | Cat# 554714 |
|  |  |  |
|  |  |  |
| Methanol | Wako | Cat# 137-01823 |
| Hydrogen peroxide | Kishida chemical | Cat# 000-37655 |
| TWEEN® 20 | Sigma-Aldrich | Cat# P7949 |
| Instant citrate buffer | LSI medience | Cat# RM102-C |
| Blocking one Histo | nacalai tesque | Cat# 06349-64 |
| Signal Enhancer HIKARI for Immunostain Solution A | nacalai tesque | Cat# 02373-54 |
| Signal Enhancer HIKARI for Immunostain Solution B | nacalai tesque | Cat# 02375-34 |
| N-Histofine® Simple Stain MAX-PO(R) | Nichireibiosciences | Cat# 414311 |
| N-Histofine® Simple Stain MAX-PO(Rat) | Nichireibiosciences | Cat# 414341F |
| ImmPACT® AMEC Red Substrate Kit, Peroxidase | Vector Laboratories | Cat# SK-4285 |
| Dako REAL™ EnVision™ Detection System, Peroxidase/DAB, Rabbit/Mouse, HRP | Dako | Cat# K5007 |
| Malinol 750cps | Muto Kagaku | Cat# 2009-2 |
| MOUNT-QUICK AQUEOUS | DAIDO SANGYO | Cat# DM-02 |
